# Role of Trace Elements in Diabetic Retinopathy: A Systematic Review

**DOI:** 10.7759/cureus.94461

**Published:** 2025-10-13

**Authors:** Niranjan Gopal, Deepa Telgote, Mustakiz Z Manir, Anjali S, Utsav Haldar, Dnyanesh Amle

**Affiliations:** 1 Biochemistry, All India Institute of Medical Sciences, Nagpur, Nagpur, IND

**Keywords:** chromium, diabetic retinopathy, iron, magnesium, manganese, oxidative stress, selenium, trace elements, type 2 diabetes mellitus, zinc

## Abstract

Diabetic retinopathy (DR) is a leading cause of vision impairment worldwide and a major microvascular complication of type 2 diabetes mellitus (T2DM). While chronic hyperglycemia remains the principal driver of DR, growing evidence suggests that dysregulation of essential trace elements, including magnesium, zinc, manganese, chromium, selenium, and iron, may contribute to retinal microvascular injury through oxidative stress and impaired endothelial function. This systematic review was conducted in accordance with the Preferred Reporting Items for Systematic Reviews and Meta-Analyses (PRISMA) 2020 guidelines, with a comprehensive search of PubMed, Scopus, Embase, Web of Science, Cochrane Library, and Google Scholar for studies published between January 2000 and June 2024. Search terms included "diabetic retinopathy," "trace elements," "magnesium," "zinc," "manganese," "chromium," "selenium," and "iron." Eligible studies comprised observational studies (cross-sectional, case-control, cohort), randomized controlled trials, and meta-analyses reporting serum, plasma, or dietary levels of trace elements in patients with T2DM, with and without DR. Data extraction was performed independently by two reviewers with consensus resolution. A total of 15 studies met the inclusion criteria. Patients with DR consistently demonstrated lower serum levels of magnesium, zinc, manganese, and chromium compared to diabetic controls without retinopathy. Selenium exhibited a U-shaped relationship, with both low and high levels associated with increased DR risk. Dysregulation of iron, particularly deficiency and evidence of iron-driven ferroptosis, was linked to retinal hypoxia, neurodegeneration, and elevated oxidative stress. These findings demonstrate that trace element imbalances are strongly associated with the risk and progression of DR. Assessment of serum trace elements may serve as a cost-effective biomarker panel for early risk stratification. However, heterogeneity across studies and the predominance of observational designs limit causal inference, underscoring the need for well-designed prospective cohorts and randomized controlled trials to determine whether targeted micronutrient supplementation can reduce DR incidence or delay progression.

## Introduction and background

Type 2 diabetes mellitus (T2DM) is a chronic metabolic disorder characterized by impaired insulin secretion from pancreatic β-cells and reduced insulin sensitivity, leading to persistent hyperglycemia [1]. It is strongly associated with obesity, sedentary lifestyle, and aging, and is perpetuated by complex genetic, environmental, and inflammatory interactions that culminate in insulin resistance and β-cell dysfunction [1,2].

T2DM has emerged as a global public health crisis, with an estimated 463 million adults affected in 2019, projected to rise to 700 million by 2045, a 51% increase [2]. The global prevalence stands at 8.8%, with slightly higher rates in men (9.6%) than women (9.0%), and the burden is disproportionately high in low- and middle-income countries [2,3]. India ranks second only to China, with 77 million cases in 2019, expected to escalate to 134 million by 2045 [2]. Alarmingly, nearly 57% of diabetes cases remain undiagnosed in India, contributing to late detection of complications and higher morbidity [2,4].

Diabetic retinopathy (DR): a growing concern

DR is a leading microvascular complication of diabetes and one of the foremost causes of vision impairment and blindness worldwide, particularly in the working-age population [5]. Chronic hyperglycemia initiates a cascade of biochemical disturbances, including oxidative stress, polyol pathway activation, protein kinase C signalling, and advanced glycation end-product accumulation, resulting in progressive microvascular damage, capillary leakage, neovascularization, and retinal neurodegeneration [3,6].

Globally, approximately 30-40% of individuals with T2DM develop some degree of DR. By 2045, it is projected that 161 million people will have DR, including 45 million with vision-threatening DR (VTDR) [4,5]. In India, a nationally representative survey reported a DR prevalence of 12.5% and VTDR prevalence of 4% among adults with diabetes aged ≥40 years [4,7,8]. This translates to nearly 3 million individuals with VTDR, highlighting the urgent need for improved risk stratification and early intervention strategies.

Emerging Role of Trace Elements in DR

Although chronic hyperglycemia is the principal driver of DR, growing evidence suggests that imbalances in essential trace elements also play a key role in its pathogenesis [9]. Micronutrients such as magnesium, zinc, manganese, chromium, selenium, and iron are crucial cofactors in enzymatic reactions regulating glucose metabolism, insulin signalling, antioxidant defence, and vascular homeostasis [10,11].

Magnesium (Mg): A cofactor in carbohydrate metabolism and insulin receptor function, hypomagnesemia is common in T2DM and linked with insulin resistance, endothelial dysfunction, and DR severity [7,12,13].

Zinc (Zn): Essential for insulin synthesis and antioxidant defense, zinc deficiency is associated with increased oxidative stress, vascular endothelial growth factor (VEGF) upregulation, and progression of DR [14,15].

Manganese (Mn): Required for mitochondrial function and manganese superoxide dismutase (MnSOD) activity, low Mn levels or MnSOD polymorphisms have been linked to higher DR risk [16,17].

Chromium (Cr): It enhances insulin receptor activity and glucose uptake; its deficiency contributes to hyperglycemia and may worsen microvascular complications [8,18,19].

Selenium (Se): Through selenoproteins such as glutathione peroxidase, selenium reduces oxidative stress and supports endothelial health, though a U-shaped association with DR risk has been reported [9,20,21].

Iron (Fe): Both deficiency and dysregulated iron metabolism may promote retinal hypoxia, oxidative injury, and ferroptosis, contributing to neurodegeneration in DR [10,22].

In countries with a high diabetes burden, such as India, regional variations in micronutrient intake and nutritional status may further influence trace element profiles and associated DR risk [23,24].

Rationale for this systematic review

Despite numerous observational studies and some clinical trials, evidence regarding the association between trace elements and DR remains fragmented. Causal relationships are unclear, and optimal supplementation strategies have not been established. Moreover, data on stage-specific associations (mild nonproliferative DR (NPDR) vs. proliferative DR (PDR) and the impact of genetic polymorphisms on trace element metabolism are limited.

Therefore, we conducted a systematic review of published literature to synthesize current evidence on the association of magnesium, zinc, manganese, chromium, selenium, and iron with DR, summarize mechanistic insights, and identify research gaps to guide future interventional studies.

## Review

Study design

This systematic review was conducted in accordance with the Preferred Reporting Items for Systematic Reviews and Meta-Analyses (PRISMA) 2020 guidelines.

Research question

The review aimed to evaluate the association of trace elements (zinc, magnesium, copper, selenium, chromium, and others) with the development and progression of DR in patients with diabetes mellitus.

Eligibility criteria

Inclusion Criteria

The inclusion criteria were as follows: studies enrolling adults aged 18 years or older diagnosed with type 1 or T2DM; those assessing serum or plasma levels of trace elements such as zinc, magnesium, copper, selenium, chromium, manganese, and iron; studies including a comparator group of diabetic patients without retinopathy or healthy controls where applicable; studies reporting an association between trace element levels and DR; and observational studies (cross-sectional, case-control, cohort), review articles, and clinical trials published in peer-reviewed journals, in English, between January 2000 and June 2025.

Exclusion Criteria

The exclusion criteria included case reports, letters to the editor, and conference abstracts without full text; studies lacking a comparator group or not reporting quantitative data on trace elements; and animal or in vitro studies.

Search strategy

A comprehensive literature search was conducted in PubMed, Scopus, Web of Science, Cochrane Library, and Google Scholar. The following combination of keywords and Medical Subject Headings (MeSH) was used: (“diabetic retinopathy” OR “DR”) AND (“trace elements” OR “zinc” OR “magnesium” OR “copper” OR “selenium” OR “chromium” OR “manganese” OR “iron”). Boolean operators AND/OR were used to refine results. Reference lists of relevant articles and reviews were manually screened to identify additional eligible studies.

Study selection

Two independent reviewers screened titles and abstracts. Full texts of potentially relevant studies were assessed for eligibility. Discrepancies were resolved by consensus or third-party adjudication.

Data extraction

Data from each included study were extracted into a predesigned MS Excel (Microsoft Corporation, Redmond, Washington, United States) sheet, recording author, year, country, study design, sample size, population characteristics, type of trace element measured, method of assessment, DR grading system used, and key results.

Quality assessment and risk of bias

The methodological quality of included studies was independently assessed by two reviewers using the Joanna Briggs Institute (JBI) critical appraisal tools appropriate for study design (cross-sectional, case-control, or cohort). Randomized controlled trials were evaluated using the Cochrane Risk of Bias 2 (RoB 2) tool. Each study was rated as having low, moderate, or high risk of bias based on criteria such as participant selection, measurement reliability, confounding control, and outcome reporting (Table 1).

**Table 1:** Quality assessment of included studies NHANES: National Health and Nutrition Examination Survey; RoB 2: Risk of Bias 2; JBI: Joanna Briggs Institute; RCT: randomized controlled trial The methodological quality of included studies was independently assessed by two reviewers using the JBI critical appraisal tools appropriate for study design (cross-sectional, case-control, or cohort). Randomized controlled trials were evaluated using the Cochrane RoB 2 tool. Each study was rated as having low, moderate, or high risk of bias based on criteria such as participant selection, measurement reliability, confounding control, and outcome reporting. Disagreements were resolved by consensus

Author	Study design	Assessment tool	Risk of bias	Key limitation
Zhu et al. [6]	Retrospective study	JBI checklist (cross-sectional)	Moderate	Limited control for confounding factors
Shivakumar et al. [7]	Cross-sectional	JBI	Moderate	Small sample size; single-center study
Alkhalidi et al. [8]	RCT (single-blind)	Cochrane RoB 2	Low	Short duration; no long-term follow-up
Chen et al. [9]	Cross-sectional (NHANES)	JBI	Low	Secondary data; possible reporting bias
Chen et al. [10]	Cross-sectional (NHANES 2005–08)	JBI	Low	Cross-sectional design limits causality
Kumar et al. [12]	Cross-sectional	JBI	Moderate	Potential selection bias; hospital-based sample
Xu et al. [13]	Cross-sectional	JBI	Moderate	No adjustment for dietary intake or duration of DM
Dascalu et al. [14]	Systematic review	JBI (systematic reviews)	Low	Heterogeneity among included studies
Miao et al. [15]	Narrative review	JBI (narrative review)	Moderate	Lack of quantitative synthesis
Zhang et al. [16]	NHANES (2011-2020)	JBI	Low	Limited to the U.S. population; self-reported data
Nugrahani et al. [17]	Meta-analysis	JBI (systematic reviews)	Low	Between-study heterogeneity
Ghosh et al. [19]	RCT (double-blinded)	Cochrane RoB 2	Moderate	Small sample; incomplete blinding
Sonkar et al. [21]	Observational	JBI	Moderate	No adjustment for confounding variables
Ganiger et al. [25]	Case-control	JBI	Moderate	Single-center; small sample
Srikanth [26]	Case-control	JBI	Moderate	Limited generalizability; small sample size

Data synthesis

**Figure 1:**
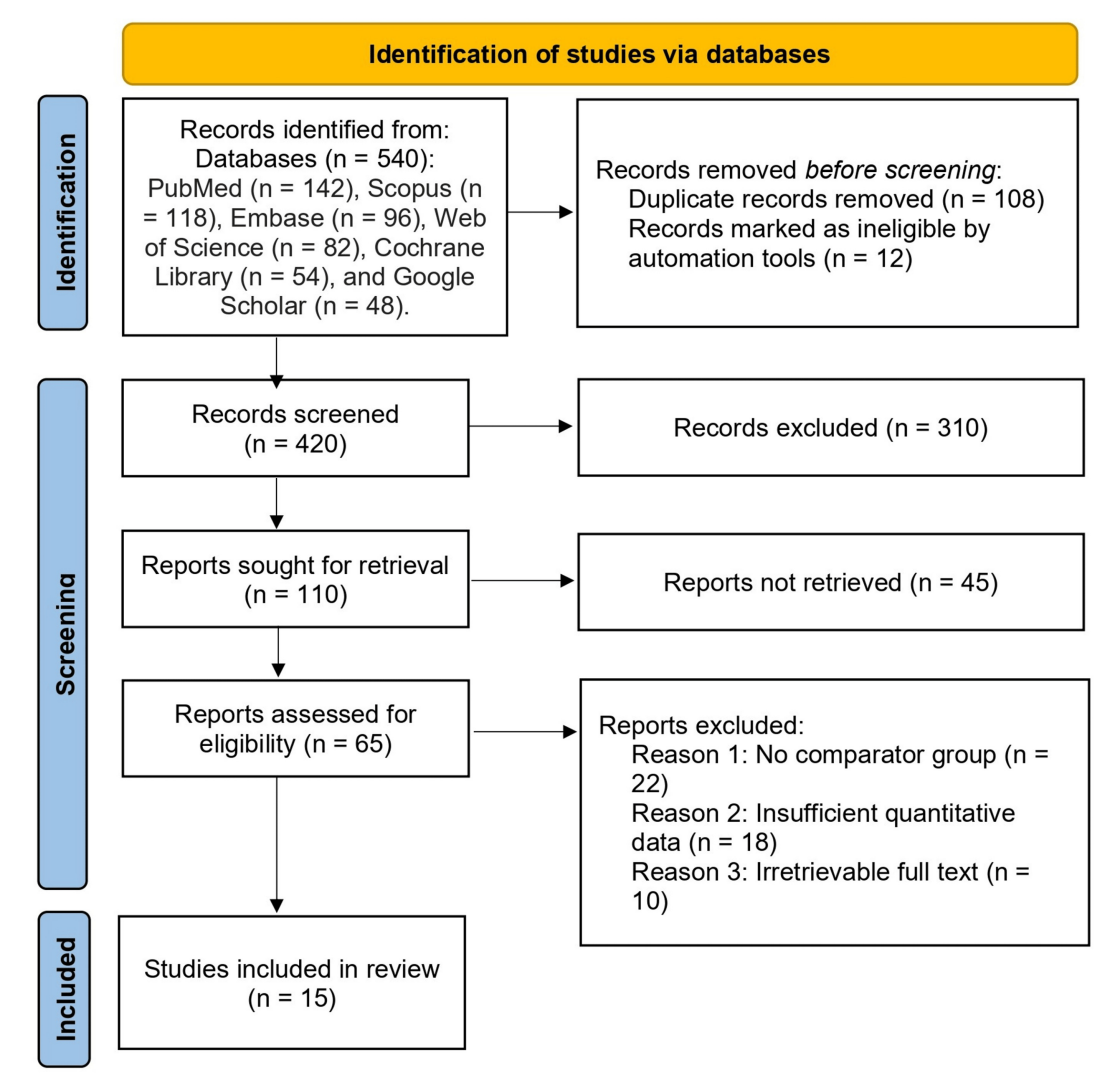
Preferred Reporting Items for Systematic Reviews and Meta-Analyses (PRISMA) flow diagram A total of 540 records were identified from the following databases: PubMed (n = 142), Scopus (n = 118), Embase (n = 96), Web of Science (n = 82), Cochrane Library (n = 54), and Google Scholar (n = 48). After removing 108 duplicate records and 12 records marked ineligible by automation tools, 420 records were screened. Of these, 310 were excluded based on title and abstract screening. A total of 110 reports were sought for retrieval, and 45 were not retrieved. A total of 65 reports were assessed for eligibility, of which 50 were excluded for the following reasons: no comparator group (n = 22), insufficient quantitative data (n = 18), and irretrievable full text (n = 10). Finally, 15 studies were included in the qualitative synthesis

A narrative synthesis was performed, summarizing associations between trace elements and DR across studies. Where possible, trends were reported (e.g., lower zinc/magnesium levels or higher copper levels in DR vs. controls). Due to heterogeneity in study design, populations, and measurement techniques, a meta-analysis was not performed (Figure 1).

Results

The systematic review collectively evaluated the association between various trace elements and DR. The included studies spanned multiple regions (India, China, the USA, and Europe) and represented diverse study designs, including cross-sectional studies, randomized controlled trials, meta-analyses, and secondary data analyses. Table 2 summarizes the key characteristics of these studies, including population details, study design, and outcomes assessed.

**Table 2:** Characteristics of included studies evaluating the association between trace elements and DR T2DM: type 2 diabetes mellitus; DR: diabetic retinopathy; NPDR: nonproliferative diabetic retinopathy; PDR: proliferative diabetic retinopathy; Mg: magnesium; Zn: zinc; Mn: manganese; Se: selenium; Cr: chromium; RCT: randomized controlled trial; NHANES: National Health and Nutrition Examination Survey; OR: odds ratio; CI: confidence interval; FBS: fasting blood sugar; PPBS: postprandial blood sugar; MnSOD: manganese superoxide dismutase (gene polymorphism) This table summarizes the study characteristics, including author and year, country of study, study design, sample size, population under investigation, trace element(s) evaluated, comparator groups, and primary outcomes measured

Author & year	Country	Study design	Sample size	Population	Trace element studied	Comparator group	Outcome measured
Zhu et al. [6]	China	Retrospective study	112	T2DM patients with and without DR & healthy controls	Zinc, manganese	DR vs non-DR vs controls	Serum Zn and Mn levels and correlation with DR
Shivakumar et al. [7]	India	Cross-sectional	104	T2DM patients with and without DR	Magnesium	DR vs No DR	Mean serum Mg level and correlation with DR severity
Alkhalidi et al. [8]	Iraq	RCT (single-blind)	60	T2DM	Chromium	Chromium supplement (200 mcg BID) + standard drug vs standard drug only	Impact of chromium supplementation on glycemic parameters
Chen et al. [9]	USA	Cross-sectional (NHANES)	645	Adults with T2DM	Selenium	DR vs non-DR	Association between Se and DR
Chen et al. [10]	USA	Cross-sectional (NHANES 2005-08)	5321	T2DM	Iron	DR vs No DR	Serum iron levels & DR prevalence
Kumar et al. [12]	India	Cross-sectional	250	T2DM patients	Magnesium	Normal Mg vs Low Mg groups	Prevalence of NPDR, PDR
Xu et al. [13]	China	Cross-sectional	239	Prediabetes & T2DM	Magnesium	Non-diabetic controls vs T2DM	Association of Mg levels with DR & other complications
Dascalu et al. [14]	Romania	Systematic review	–	T2DM patients	Zinc	DR vs non-DR vs healthy	Serum zinc levels, DR severity
Miao et al. [15]	China	Narrative review	–	T2DM patients	Zinc	DR vs non-DR	Association of zinc with DR
Zhang et al. [16]	USA	NHANES (2011-2020)	1583	T2DM patients	Manganese, selenium	DR vs No DR	Association between Mn and Se with DR
Nugrahani et al. [17]	Multi-country	Meta-analysis	2132	T2DM	Manganese (MnSOD polymorphism)	VV vs AA genotypes	DR risk (OR, 95% CI)
Ghosh et al. [19]	India	RCT (double-blind)	100	Uncontrolled T2DM	Chromium	Study group vs placebo	Effect of Cr supplementation on HbA1c, FBS, PPBS, Insulin
Sonkar et al. [21]	India	Observational	200	T2DM patients (some with DR)	Zinc, selenium, magnesium, chromium	T2DM vs healthy	Trace element levels vs glycemic control and DR presence
Ganiger et al. [25]	India	Case-control	150	T2DM patients with and without DR & healthy controls	Zinc	DR vs non-DR vs healthy	Serum Zn levels and correlation with HbA1c
Srikanth [26]	India	Case-control	190	T2DM patients with and without DR & healthy controls	Magnesium	DR vs non-DR vs controls	Serum Mg levels and correlation with DR

Magnesium

Lower serum magnesium levels were consistently associated with higher prevalence and severity of DR across multiple studies. Kumar et al. [12] reported significantly greater rates of NPDR (62.7%) and proliferative DR (21.8%) among patients with hypomagnesemia compared to those with normal magnesium levels (14.3% and 8.6%, respectively; p < 0.05). Similar results were observed in the studies by Xu et al. [13] and Shivakumar et al. [7], strengthening the evidence that hypomagnesemia may contribute to microvascular complications in diabetes.

Zinc

Zinc deficiency was commonly observed in DR patients. Dascalu et al. [14] demonstrated lower zinc levels in DR cases, particularly in those with poor glycemic control. Experimental data from Miao et al. [15] further support zinc’s protective role, showing that deficiency enhances oxidative stress, pericyte apoptosis, and pathological neovascularization, all of which are key mechanisms in DR progression.

Manganese

Evidence suggests that manganese may play a protective role against DR. Zhang et al. [16], using the National Health and Nutrition Examination Survey (NHANES) data, reported a negative association between serum manganese and DR prevalence, particularly in obese, older men. Additionally, Nugrahani et al. [17] identified a genetic risk factor, MnSOD Ala16Val polymorphism (VV genotype), that increased susceptibility to DR (p < 0.01).

Chromium

Chromium supplementation was shown to improve glycemic parameters and may lower DR risk. Alkhalidi et al. [8] observed a significant reduction in HbA1c levels from 10.4% to 7.2% after three months of chromium supplementation (p < 0.05). Ghosh et al. [19] also reported significant improvements in fasting blood glucose, postprandial glucose, HbA1c, and insulin sensitivity following chromium therapy.

Selenium

The association between selenium and DR appears to follow a U-shaped curve. Chen et al. [9] reported that both selenium deficiency and excess were linked with increased DR prevalence, with an optimal level near 106.8 µg/L. Sonkar et al. [21] found no direct association with DR but noted that selenium deficiency was correlated with poor glycemic control, potentially influencing disease progression indirectly.

Iron

Two studies highlighted the role of iron in DR pathogenesis. Chen et al. [10] demonstrated that lower serum iron levels were independently associated with higher DR prevalence, even after multivariate adjustment. Wang et al. [22] provided experimental evidence linking iron-driven ferroptosis to retinal neurodegeneration in diabetic models, suggesting a possible mechanistic pathway.

Taken together, these findings indicate that dysregulation of trace elements, particularly magnesium, zinc, manganese, chromium, selenium, and iron, plays a significant role in the development and progression of DR. The detailed summary of individual study findings is presented in Table 3.

**Table 3:** Summary of findings on trace elements and diabetic retinopathy DR: diabetic retinopathy; NPDR: nonproliferative diabetic retinopathy; PDR: proliferative diabetic retinopathy; T2DM: type 2 diabetes mellitus; T2DCON: type 2 diabetes mellitus without complications; Mg: magnesium; Zn: zinc; Mn: manganese; Se: selenium; Cr: chromium; MnSOD: manganese superoxide dismutase (gene polymorphism); HbA1c: glycated hemoglobin; FBS: fasting blood sugar; PPBS: postprandial blood sugar; BMI: body mass index; NA: not available; NS: not significant This table provides a comprehensive overview of the key findings from studies investigating the association between various trace elements and DR. Each row summarizes the author, year, major findings, and reported statistical significance

Trace element	Author (year)	Key findings	Statistical significance
Magnesium	Shivakumar et al. [7]	Serum Mg significantly lower in DR group vs. non-DR group	p = 0.029
	Kumar et al. [12]	Hypomagnesemia group had higher prevalence of NPDR (62.7%) and PDR (21.8%) vs. normal Mg group (14.3% and 8.6%)	p < 0.001
	Xu et al. [13]	Serum Mg levels significantly lower in DR group compared to T2DM without complications (T2DCON)	p < 0.01
	Srikanth MP [26]	Serum Mg significantly lower in DR group vs. non-DR group	p = 0.022
	Sonkar et al. [21]	No direct association between Mg and DR, but deficiency worsened glycemic control	NA
Zinc	Zhu X et al. [6]	Zinc levels significantly lower in DR group compared to DM group	p < 0.05
	Dascalu et al. [14]	Zinc levels lower in DR patients, correlated with poor glycemic control	NA
	Miao et al. [15]	Zinc deficiency worsens oxidative stress, promotes pericyte apoptosis and retinal neovascularization	NA
	Sonkar et al. [21]	No direct association between Zn and DR, but deficiency worsened glycemic control	NA
	Ganiger A et al. [25]	Strong negative correlation between serum zinc and HbA1c in retinopathy cases	p < 0.001
Manganese	Zhu X et al. [6]	Mn levels significantly lower in the DR group compared to the DM group	p < 0.05
	Nugrahani et al. [17]	MnSOD Ala16Val polymorphism (VV genotype) significantly increased DR risk	p < 0.0001
	Zhang et al. [16]	Inverse association between serum Mn levels and DR prevalence; strongest in older men with BMI ≥ 30	p = 0.015
Chromium	Alkhalidi et al. [8]	Chromium supplementation reduced HbA1c from 10.4% to 7.2% in 3 months	p = 0.001
	Ghosh et al. [19]	Chromium supplementation improved FBS, PPBS, HbA1c, and insulin sensitivity	p < 0.0001
	Sonkar et al. [21]	No direct association between chromium and DR, but deficiency worsened glycemic control	NA
Selenium	Chen X et al. [9]	U-shaped relationship between Se and DR; optimal level ≈ 106.8 µg/L	Significant nonlinear association
	Zhang et al. [16]	No association between serum Se levels and DR	p = 0.125 (NS)
	Sonkar et al. [21]	No direct association between Se and DR, but Se deficiency worsened glycemic control	NA
Iron	Chen et al. [10]	Lower serum iron levels significantly associated with higher DR prevalence	p < 0.004
	Wang et al. [22]	Ferroptosis (iron-driven cell death) linked to retinal neurodegeneration in diabetic rats	Preclinical experimental evidence

Discussion

DR remains one of the most significant microvascular complications of diabetes mellitus and a leading cause of vision loss worldwide. The current systematic review synthesizes available evidence on the role of trace elements in the pathogenesis and progression of DR, providing important insights into their potential utility as biomarkers or therapeutic targets. Our review found consistent evidence linking hypomagnesemia with increased risk and severity of DR. Kumar et al. [12] reported that patients with hypomagnesemia exhibited markedly higher prevalence of NPDR and PDR compared to those with normal magnesium levels. These findings were corroborated by Xu et al. [13] and Shivakumar et al. [7], suggesting that magnesium deficiency may exacerbate microvascular dysfunction through mechanisms such as impaired endothelial function, increased oxidative stress, and altered glucose metabolism.

Zinc, another critical micronutrient, was also found to be significantly lower in patients with DR. Dascalu et al. [14] observed that zinc levels inversely correlated with DR severity, particularly in those with poor glycemic control, while Miao et al. [15] demonstrated that zinc deficiency promotes oxidative stress and pericyte apoptosis, processes central to DR pathogenesis. These results underscore zinc’s role as a potent antioxidant and a cofactor for numerous enzymes involved in retinal protection.

The relationship between manganese and DR has been explored less extensively but appears to suggest a protective effect. Zhang et al. [16] reported a negative association between manganese levels and DR prevalence, with the strongest association seen in older men with obesity, possibly reflecting manganese’s role in antioxidant defence through the MnSOD pathway. This is further supported by Nugrahani et al. [17], who showed that the MnSOD Ala16Val polymorphism (VV genotype) increased DR susceptibility, indicating a gene-environment interaction that warrants further exploration.

Chromium supplementation studies showed improvements in glycemic control, with Alkhalidi et al. [8] reporting significant reductions in HbA1c levels, while Ghosh et al. [19] demonstrated improvements in multiple metabolic parameters. Given that poor glycemic control is a key risk factor for DR, chromium’s role in enhancing insulin sensitivity may indirectly reduce DR risk. Selenium displayed a more complex, nonlinear relationship with DR, as reported by Chen et al. [9], who demonstrated a U-shaped curve with both low and high selenium levels associated with increased DR prevalence. This finding highlights the need for maintaining optimal selenium status rather than indiscriminate supplementation. Iron dysregulation was also implicated, with Chen et al. [10] reporting lower serum iron levels in DR patients, while Wang et al. [22] demonstrated that iron-driven ferroptosis may contribute to retinal neurodegeneration in diabetic models, suggesting a potential mechanistic link between iron metabolism and retinal damage.

Despite these important findings, our review has limitations. The majority of included studies were cross-sectional, limiting causal inference. There was heterogeneity in study design, sample size, and methods of trace element measurement, which precluded meta-analysis. Additionally, few interventional studies were available, making it difficult to determine whether correcting trace element deficiencies would translate into clinically meaningful reductions in DR incidence or severity. Future well-designed prospective studies and randomized controlled trials are needed to validate these associations and explore therapeutic implications.

## Conclusions

This systematic review highlights the significant association between essential trace elements magnesium, zinc, manganese, chromium, selenium, and iron and the development and progression of DR in patients with T2DM. The evidence suggests that deficiencies in magnesium, zinc, manganese, and chromium, as well as dysregulation of selenium and iron, may contribute to oxidative stress, endothelial dysfunction, and microvascular injury, thereby accelerating retinal damage. These findings underscore the potential of trace element assessment as a cost-effective tool for early risk stratification and targeted preventive interventions, particularly in resource-limited settings with a high diabetes burden, such as India. However, given the heterogeneity of available studies and the predominance of observational designs, further large-scale, prospective studies and randomized controlled trials are warranted to establish causality and clarify whether supplementation or correction of these deficiencies can translate into reduced incidence or severity of DR.
